# Race against dengue

**DOI:** 10.7554/eLife.96018

**Published:** 2024-02-15

**Authors:** Swee Sen Kwek, Eng Eong Ooi

**Affiliations:** 1 Department of Infectious Diseases, Singapore General Hospital Singapore Singapore; 2 Duke-NUS Medical School Singapore Singapore; 3 Viral Research and Experimental Medicine Centre, SingHealth Duke-NUS Academic Medical Centre Singapore Singapore; 4 Department of Translational Clinical Research, Singapore General Hospital Singapore Singapore; 5 Saw Swee Hock School of Public Health, National University of Singapore Singapore Singapore

**Keywords:** Dengue, virus, vaccine, viremia, drugs, Other

## Abstract

Understanding the kinetics of dengue viruses in the bloodstream can provide insights into the clinical outcomes of the disease.

**Related research article** Vuong NL, Quyen NTH, Tien NTH, Kien DTH, Duyen HTL, Lam PK, Tam DTH, Ngoc TV, Jaenisch T, Simmons CP, Yacoub S, Wills BA, Geskus RB. 2024. Dengue viremia kinetics and effects on platelet count and clinical outcomes: An analysis of 2340 patients from Vietnam. *eLife* **13**:RP92606. doi: 10.7554/eLife.92606.

Dengue fever is an acute viral disease that afflicts an estimated 100 million people each year, killing around 40,000 of those infected (Bhatt et al., 2013). It can be caused by infection with any of the four different dengue viruses, so it is possible to catch the disease four times. Moreover, a primary infection can be followed by a secondary infection and, in some cases, post-secondary infections.

The geographic footprint of *Aedes aegypti*, the mosquito that transmits dengue viruses, covers the tropics and is expanding both northwards – it can now be found in northern California – and southwards (Pless et al., 2017). Globalization, urbanization and travel are the main drivers for the spread of the disease, which is undoubtedly going to worsen with climate change (Messina et al., 2019). There are currently no licensed antiviral drugs to treat dengue, and the two vaccines that have been licensed are of limited benefit, so as we wait for better vaccines and drugs, there is an urgent need to find new ways to combat dengue to complement existing efforts that target *Aedes aegypti*.

Dengue develops in phases. In the first two to three days after the onset of fever, dengue presents with symptoms similar to influenza and other viral infections. Around four to six days after onset, more dengue-specific symptoms – such as pain at the back of the eyes and decreased platelet count – become apparent. The critical phase of the illness occurs as the fever subsides: the risk of inflammation-driven vascular damage and leakage increases, and if this is not effectively managed, the result can be dangerously low blood pressure and multi-organ failure. When this happens, the fatality rate can be as high as 30%.

Higher levels of the viruses in the blood (also known as viremia) are thought to increase the severity of the disease. Lowering the amount of dengue virus in the bloodstream may, therefore, reduce the risk of severe dengue (Low et al., 2017). Now, in eLife, Nguyen Lam Vuong, Ronald Geskus and colleagues report how an antiviral drug might be able to alter the course of disease progression in dengue patients (Vuong et al., 2024). They also highlight the challenges that drug developers face.

Using pooled data from three studies conducted in Vietnam between 2000 and 2016, which included daily platelet counts and measurements of viremia for 2,340 dengue patients, Vuong et al. were able to reconstruct the kinetics of the different dengue viruses. In particular, the researchers were able to determine which type of dengue virus had been present and whether patients were experiencing a primary or secondary infection. Although they did not attempt to distinguish between secondary and post-secondary infections, most of the secondary dengue patients were likely experiencing their second infection, as the third and fourth infections are mostly asymptomatic (Olkowski et al., 2013).

The results of Vuong et al. – who are based at the Oxford University Clinical Research Unit Vietnam and other research institutes in Vietnam, the United Kingdom, the United States, Germany and Australia – show that viremia levels decreased rapidly following the onset of symptoms, depending on the type of virus, with dengue virus type-1 having the highest viremia levels in the first five to six days. High levels of viremia in the first two days following onset also correlated with a higher risk of severe dengue and risk of vascular leakage. In both primary and secondary infections, viremia levels of all four viruses decreased significantly after the first two days of fever, in correlation with the severity of the disease.

The results indicate that lowering viral levels could thus reduce the risk of vascular leakage and severe dengue. However, antiviral drugs would need to be highly potent in targeting viral replication to reduce viral levels faster than their natural decay rate. Antiviral treatment would likely also need to be given within the first two days following fever onset to effectively alter the course of the illness.

The ability to initiate treatment early also requires an early diagnosis, but it is difficult to differentiate between dengue and other viral fevers during the early stages of illness. Current point-of-care rapid tests detect the viral protein NS1 in the blood of dengue patients. However, this protein is less detectable in those with secondary infections (Chaterji et al., 2011): this is unfortunate because secondary infection is associated with a greater risk of severe dengue. This means that the use of current tests could thus, paradoxically, exclude many patients who need antiviral treatment.

To effectively target dengue fever, there is a need to develop both new drugs and new tests. While there are currently three highly promising drugs being tested in phases II and III of clinical trials, efforts to develop a low-cost rapid test that can accurately diagnose both primary and secondary infection are lacking. It would be a tragedy if – once licensed – antiviral drugs could not be given to dengue patients early enough to alter disease progression due to the lack of a suitable early diagnostic test.
